# A Dynamic Price Game Model in a Low-Carbon, Closed-Loop Supply Chain Considering Return Rates and Fairness Concern Behaviors

**DOI:** 10.3390/ijerph16111978

**Published:** 2019-06-04

**Authors:** Qiuxiang Li, Mengnan Shi, Yimin Huang

**Affiliations:** 1 Institute of management science and engineering, Henan University, Kaifeng 475004, China; lqxkycg@henu.edu.cn; 2 Business School, Henan University, Kaifeng 475004, China; 104754181123@vip.henu.edu.cn; 3 School of Management & Economics, North China University of Water Resources and Electric Power, Zhengzhou 450046, China

**Keywords:** price game, CER, return rate, complexity

## Abstract

In this paper, we developed a dynamic price game model for a low-carbon, closed-loop supply chain system in which (1) the manufacturer had fairness concern and carbon emission reduction (CER) behaviors, and market share and profit maximization were their objectives, and (2) the retailer showed fairness concern behaviors in market competition and provided service input to reduce return rates. The retailer recycled old products from customers, and the manufacturer remanufactured the recycled old products. The effects of different parameter values on the stability and utility of the dynamic price game model were determined through analysis and numerical simulation. Results found that an increasing customer loyalty to the direct marketing channel decreased the stable region of the manufacturer’s price adjustment and increase that of the retailer. The stable region of the system shrank with an increase of CER and the retailer’s service level, which expanded with return rates. The dynamic system entered into chaos through flip bifurcation with the increase of price adjustment speed. In the chaotic state, the average utilities of the manufacturer and retailer all declined, while that of the retailer declined even more. Changes to parameter values had a great impact on the utilities of the manufacturer and retailer. By selecting appropriate control parameters, the dynamic system can return to a stable state from chaos again. The research of this paper is of great significance to participants’ price decision-making and supply chain operation management.

.

## 1. Introduction

With increasingly serious global environmental problems, low-carbon products for environmental protection have become an important direction in sustainable development. Both the government and consumers are calling for green production and low-carbon supply. The report of the Nineteenth National Congress of the Communist Party of China points out that high-quality development is to meet the growing needs of people for a better life and to normalize green development. At present, many manufacturers have made ecodesigns, carbon emission reductions (CERs), and green development through technology and product design [1,2].

Scholars have conducted research on low-carbon issues. Liu et al. [3] analyzed the impact of income inequality on carbon emission in the US and found that income inequality exacerbates carbon emissions in the US in the short term, while it promotes carbon emission reductions in the long run. Zhang et al. [4] found that the probability of the manufacturer introducing green technology was negatively correlated with the cost of government intervention, and it was positively correlated with punishment by the government for the manufacturer’s speculative behavior. Considering both economic and energy goals, Ye et al. [5] established a dual-objective programming model that integrated 3E goals with quota allocation issues. They found the 3E approach was better than the ancestral approach in some respects in the case of Guangdong province. Tran et al. [6] analyzed the relationship between an emissions trading scheme and various revenue recycling options using the case of Australian households. Results showed that Australia’s real GDP contracted slightly because of emission permit prices. Lin and Jia [7] found emission trading mechanism fines had significant impacts on the cost of enterprise emission trading mechanisms, commodity prices, energy-intensive industrial output, GDP losses, and carbon dioxide emission reduction, but they had a small impact on the intensity of carbon emissions and emission reduction costs. Liu [8] developed four common cost-sharing models in a low-carbon supply chain, analyzed its pricing rules, and used a revenue sharing contract to coordinate the supply chain. Lou and Ma [9] studied the effect of sales efforts and CER efforts on the complexity of the supply chain system and found that price adjustment parameters had a greater impact on stability and profits of supply chain system than sales efforts and carbon emission reductions. Hui et al. [10] considered a low-carbon, closed-loop supply chain and examined optimal decisions and performances in different situations (competitive pricing and competitive low-carbon promotion). Yang et al. [11] considered two competitive supply chains under the cap and trade scheme in vertical and horizontal directions, and they found that there was a higher CER rate and a lower retail price in vertical cooperation. Liu et al. [12] investigated the impacts of fairness concerns on the production sustainability level, low-carbon promotion level, and profitability of the supply chain based on incomplete rationality behavior. Few papers studied the simultaneous effects of CER, return rates, and service input on price determination in a closed-loop supply chain.

With the rapid development of e-commerce, the coexistence of online sales channels and traditional sales methods has gradually become a common business model, and the return rates are also on the rise. Many scholars have made keen observations and conducted research on the phenomenon of returns. Crocker and Letizia [13] found that the retailer may take concealed actions to reduce the expected sales of products; the best policy for the retailer for return goods is that the manufacturer requires the retailer to pay full wholesale payment. Zheng et al. [14] examined how product return rates of the reverse chain influenced the determination of market price and profits of two supply chains in different competition structures. Giri and Sharma [15] found that, under the condition of sequential optimization, the acceptable quality levels of retailers’ returns were lower than those of global optimization, and integration of supply chain members led to a decrease in the number of deliveries from manufacturers to retailers. From the perspective of consumer utility function, Ofek et al. [16] studied the problems of multichannel consumer returns, retailer service, and pricing under the risk of return goods. Ramanathan [17] found that, through rating data of online consumers, the performance of enterprises in dealing with returns could affect customer loyalty. 

Fairness concerns are another important behavior factor that enterprises pay attention to. Studies about fairness concerns showed that it had significant influence on price strategies and efficiency of supply chains [18,19]. Wang et al. [20] studied decision-making and coordination of an e-commerce supply chain with manufacturer’s fairness concerns. Li et al. [21] found the market shares of retailers were related to the impact of manufacturer fairness concerns on retailer profits. Niu et al. [22] analyzed the supplier’s decision of whether to open an online direct channel or not by incorporating channel power and fairness concerns. They found that supplier fairness concerns effectively reduced their incentives to open an online channel. Ma et al. [23] studied the pricing decisions of a closed-loop supply chain considering market efforts and fairness concerns. Du et al. [24] indicated that fairness concerns could promote and coordinate the supplier and manufacturer to invest more in sustainable development of green technology innovations. Liang and Qin [25] developed an estimation game model by fuzzy theory with fuzzy fairness concerns.

Some researchers studied dynamic characteristics of the supply chain. Puu [26] briefly analyzed three oligopoly competition conditions and pointed out that strange attractors could appear in the duopoly model. Matouk et al. [27] analyzed the stability of a discrete-time dynamical game model and discussed the Neimark–Sacker bifurcation Huang et al. [28] investigated the influences of parameters on the stability of three dynamic game models, with the risk-averse manufacturer providing the complementary product. Wu and Ma [29] found that the game model introduced chaos in two ways: flip fluctuation and Neimark–Sacker bifurcation. Huang and Li [30] found the probabilistic selling supply chain system would introduce chaos from the higher system’s entropy through flip bifurcation or Neimark–Sacker bifurcation. Li and Ma [31] analyzed system stability affected by the customer risk aversion and the customer preference for probabilistic products. The papers mentioned above provided many methods and perspectives for analyzing complex characteristics of the supply chain.

However, few papers simultaneously considered customer’s behavior for return goods, retailer’s service, and manufacturer’s behavior for CER in a closed-loop supply chain. It is a very interesting topic to study the influence of return rates, the service level of the retailer, and CER of the manufacturer on the price and stability of a low-carbon, closed-loop supply chain system.

This study can be considered as an extension of the work of Zhang et al. [32]. They set up the demand function considering price difference and return risk; however, their research did not consider the impact of CER and service input on the demand function. This paper considers more realistic factors affecting the demand of low-carbon products (i.e., serves inputs, return rate, and CER) and focuses on complex analysis of a low-carbon, closed-loop supply chain.

Our theoretical contribution is as follows. The first contribution is to construct a low-carbon, closed-loop supply chain model, in which the demand function is affected by sales efforts, return rates, and CER. The second contribution is to study the stability and profitability of the low-carbon, closed-loop supply chain system under a dynamic game structure.

This paper is organized as follows. The dynamic game model of a low-carbon, closed-loop supply chain is developed in Section 2. In Section 3, the local stability of the dynamic game model is given using analytical analysis and numerical simulation. The complexity analysis of the dynamic game model is investigated in Section 4. Section 5 gives the global stability analysis of the dynamic game model using basins of attraction. Section 6 discusses chaos control of the dynamic game model. Section 7 gives conclusions.

## 2. Model Description

### 2.1. Basic Model Description

**Figure 1:**
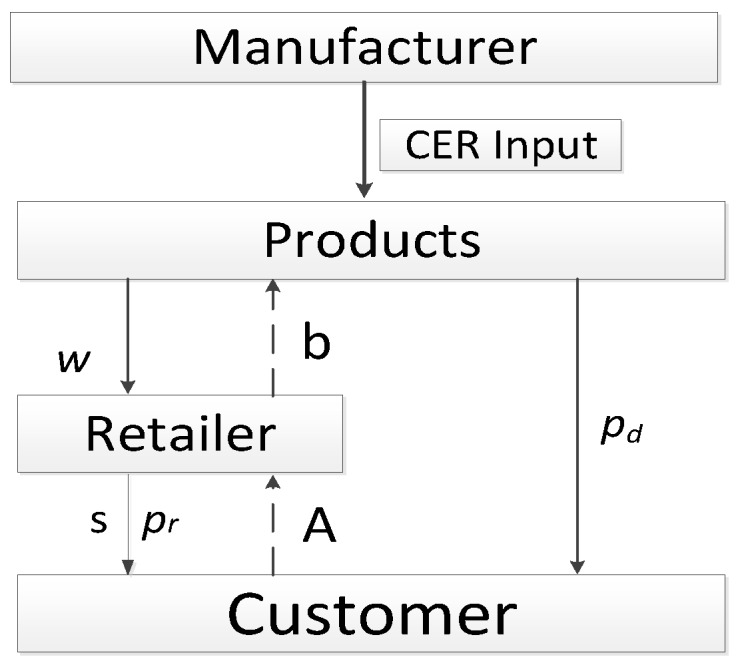
The low-carbon, closed-loop supply chain system.

In this paper, we considered a low-carbon, closed-loop supply chain consisting of a manufacturer, a retailer, and consumers (as shown in Figure 1). There were two sales channels in the forward supply chain, one was that the manufacturer sold the products to the retailer at wholesale price (w) and the retailer sold products to consumers at price $p_{r}$, and the other was that the manufacturer developed the direct marketing channel to sell products to the consumer at price $p_{d}$. In the reverse supply chain, the retailer recycled the old products from customers at unit price A and sold them to the manufacturer at unit price b, and the manufacturer remanufactured recycled old products. The manufacturer made market share and profit maximization as their objectives, while they had fairness concern behaviors and low-carbon behaviors. The retailer showed fairness concern behaviors in market competition and provided service input to reduce return rates.

### 2.2. Model Hypothesis

(1) Assuming that the unit cost of the manufacturer producing a new product is c, the unit saving cost of the manufacturer producing a remanufactured product is x, then the production cost of the remanufacturing product is c−x. In order to ensure the practical significance of this study, the following condition should be met: A<b<x. That is, the manufacturer’s transfer payment price is higher than the unit recovery price of the retailer but lower than the unit saving cost brought by remanufacturing. Under this condition, the manufacturer and retailer had the incentive to recycle and remanufacture each other. Assuming that there was no difference in quality, function, and utility between new products and remanufactured products, consumers had the same acceptance for the two kinds of products.

(2) Assuming the recovery rate of used products is τ(0≤τ≤1), the average unit production cost of the manufacturer can be expressed as $\bar{c}=\left(1-\tau \right)c+\tau \left(c-x\right)=c-\tau x$. All recycled old products are used for remanufacturing, regardless of the situation that remanufacturing is constrained by the quantity of recycled products. The retailer’s total cost of recovery consists of fixed cost and variable cost.

(1) $$C_{1}\left(\tau \right)=f\left(\tau \right)+\tau \left(b-A\right)\left(D_{r}+D_{d}\right),$$

where $f\left(\tau \right)=\frac{1}{2}h_{1}\tau^{2},\;h_{1}>0$ is a fixed investment for the retailer to recycle products, and $D_{r}$ and $D_{d}$ indicate consumer demands in the traditional channel and direct channel, respectively.

(3) Assuming that there is the same return rate (r) for direct marketing channels and traditional channels when the retailer has not provided sales services, the return rate of the traditional channel after the retailer provides sales service is $r_{r}=\left(1-s\right)r$, where s is the service level of traditional channel provided by the retailer.

(4) The retailer can only send orders to the manufacturer through the traditional channel and cannot order directly from the manufacturer through the direct marketing channel [33].

### 2.3. Profit Functions

Market demand is affected, among other factors, by product price, return rate, service level, and CER level. Assuming that market demand is a linear function of product price, return rates, service level, and CER level.

(2) $$\{\begin{array}{l} D_{d}=\rho a-b_{1}p_{d}+c_{1}p_{r}-e_{d}r+e_{rd}\left(1-s\right)r+\beta dk\\ D_{r}=\left(1-\rho \right)a-b_{2}p_{r}+c_{2}p_{d}-e_{d}\left(1-s\right)r+e_{dr}r+\left(1-\beta \right)dk \end{array},$$

where a is the basic demand of the market; ρ∈(0, 1) is the customer’s loyalty to the direct channel; β∈(0, 1) is the customer’s loyalty to the demand caused by CER; $b_{i},\;i=1,2$ is the price elasticity coefficient; $c_{i}$ is the cross-price elasticity coefficient (i=1,2); $e_{d}$ is the return rate sensitivity coefficient; and $e_{rd}$ and $e_{dr}$ are the cross-return rate sensitivity coefficients, where d is the sensitivity of the demand for CER behavior.

The costs of CER and serve input are as follows:

(3) $$\{\begin{array}{c} C_{k}=\frac{\varepsilon k^{2}}{2}\\ C_{s}=\frac{h_{2}s^{2}}{2} \end{array}.$$

where ε is the cost coefficient of CER of products, and $h_{2}\left(h_{2}>0\right)$ is the cost coefficient of serviceinput.

The profits of the manufacturer and retailer are as follows:

(4) $$\{\begin{array}{l} \pi_{m}=\left(w-c+s\tau \right)D_{r}+\left(p_{d}-c+s\tau \right)D_{d}-b\tau \left(D_{d}+D_{r}\right)-\frac{\varepsilon k^{2}}{2}\\ \pi_{r}=\left(p_{r}-w\right)D_{r}+\tau \left(b-A\right)\left(D_{r}+D_{d}\right)-\frac{1}{2}h_{1}\tau^{2}-\frac{1}{2}h_{2}s^{2} \end{array}.$$

Inspired by literatures [34,35], the utility functions of the manufacturer and retailer can be constructed as follows:

(5) $$\{\begin{array}{l} U_{m}=\mu \pi_{m}+\left(1-\mu \right)\frac{p_{d}D_{d}}{p_{d}D_{d}+p_{r}D_{r}}-\lambda_{2}\left(\pi_{r}-\gamma \pi_{mt}\right)\\ U_{r}=\pi_{r}-\lambda_{1}\left(\pi_{mt}-\pi_{r}\right) \end{array},$$

where $\lambda_{1}\;\mathrm{and}\;\lambda_{2}\in \left(0,\;1\right)$ represent the coefficients of fairness concern of the manufacturer and retailer, γ∈(0,1) denotes the relative profit coefficient, and μ∈(0,1) denotes the balance coefficient of the manufacturer between the profit and the market share of the manufacturer. $p_{d}D_{d}$ is the manufacturer’s sales revenue from the direct marketing channel, and $p_{r}D_{r}$ is the retailer’s sales revenue from the traditional sale channel. $\pi_{mt}$ is the manufacturer’s profit from the traditional sale channel, where the retailer compares its absolute profit with the profit of the manufacturer from traditional sale channel. The manufacturer only compares the relative profit from the traditional sale channel with the profit of the retailer in the traditional sale channel.

Because the manufacturer’s sales revenue has the same change trend with market share, we can replace market share with sales revenue [36,37]:

(6) $$\{\begin{array}{l} U_{m}=\mu \pi_{m}+\left(1-\mu \right)p_{d}D_{d}-\lambda_{2}\left(\pi_{r}-\gamma \pi_{mt}\right)\\ U_{r}=\pi_{r}-\lambda_{1}\left(\pi_{mt}-\pi_{r}\right) \end{array}.$$

Substituting the Equations (1), (2), and (3) into Equation (6), the marginal utilities of the manufacturer and retailer can be obtained by taking the first-order partial derivatives of $U_{m}$ and $U_{r}$ on $p_{d}$ and $p_{r}$, respectively.

$\frac{\partial U_{m}}{\partial p_{d}}=dk\beta +a\rho -re_{d}+re_{rd}-rse_{rd}+c_{1}p_{r}+b_{1}\left[\mu \left(c+b\tau -x\tau \right)-2p_{d}+\left(-A+b\right)\tau \lambda_{2}\right]+$$\mu \left(-c+w-b\tau +x\tau \right)+c_{2}\lambda_{2}\left[w-c\gamma +w\gamma +A\tau -b\tau +x\gamma \tau -p_{r}\right)]$.

$\frac{\partial U_{r}}{\partial p_{r}}=\left(1+\lambda_{1}\right)\left[a\left(1-\rho \right)+dk\left(1-\beta \right)+\tau c_{1}\left(b-A\right)+sre_{d}\right]+c_{2}\left(1+\lambda_{1}\right)p_{d}+b_{2}\left(w+A\tau -b\tau \right)+$$\lambda_{1}b_{2}\left[-c+2w+\tau \left(A-b+x\right)\right]-2b_{2}\left(1+\lambda_{1}\right)p_{r}$.

By solving $\frac{\partial U_{m}}{\partial p_{d}}=0,\;\frac{\partial U_{r}}{\partial p_{r}}=0,$ we can get the best decisions of the manufacturer and retailer:

$$\begin{array}{l} p_{d}^{*}=m_{0}+\frac{2b_{1}\left(c_{1}-c_{2}\lambda_{2}\right)\left\{wb_{2}+A\tau b_{2}-b\tau b_{2}+\left(b_{2}\lambda_{1}\left[-c+2w+\tau \left(A-b+x\right)\right]+\left(1+\lambda_{1}\right)(m_{1}-m_{5}\right)\right\}}{2b_{1}\left(1+\lambda_{1}\right)\left[4b_{1}b_{2}+c_{2}\left(-c_{1}+c_{2}\lambda_{2}\right)\right]}+\\ \frac{m_{4}\left[dk\beta +a\rho +\mu b_{1}\left(c+b\tau -x\tau \right)+\mu c_{2}m_{3}-r(e_{d}-e_{\mathrm{rd}}+se_{\mathrm{rd}}\right)+\tau b_{1}\lambda_{2}\left(-A+b\right)+c_{2}\lambda_{2}m_{2}]}{2b_{1}\left(1+\lambda_{1}\right)\left[4b_{1}b_{2}+c_{2}\left(-c_{1}+c_{2}\lambda_{2}\right)\right]};\\ p_{r}^{*}=\frac{2b_{1}\left(1+\lambda_{1}\right)\left[a+dk-dk\beta -a\rho +re_{\mathrm{dr}}+\tau c_{1}\left(b-A\right)-re_{d}\left(1-s\right)\right]}{\left(1+\lambda_{1}\right)\left[4b_{1}b_{2}+c_{2}\left(-c_{1}+c_{2}\lambda_{2}\right)\right]}-\frac{2b_{1}b_{2}\left[w+\tau \left(A-b\right)-\lambda_{1}\left(c-2w-A\tau +b\tau \right)\right]}{\left(1+\lambda_{1}\right)\left[4b_{1}b_{2}+c_{2}\left(-c_{1}+c_{2}\lambda_{2}\right)\right]}+\\ \frac{c_{2}\left(1+\lambda_{1}\right)\left[dk\beta +a\rho -re_{d}+re_{\mathrm{rd}}-rse_{\mathrm{rd}}+\mu b_{1}\left(c+b\tau -s\tau \right)+\tau b_{1}\lambda_{2}\left(-A+b\right)+\mu c_{2}\left(-c+w-b\tau +s\tau \right)+c_{2}\lambda_{2}\left(w-c\gamma +w\gamma +A\tau -b\tau \right)\right]}{\left(1+\lambda_{1}\right)\left[4b_{1}b_{2}+c_{2}\left(-c_{1}+c_{2}\lambda_{2}\right)\right]}. \end{array}$$

where $m_{0}=\frac{1}{2b_{1}}\{dk\beta +a\rho +\mu b_{1}\left[c+\tau \left(b-s\right)\right]+\mu c_{2}\left[-c+w+\tau \left(-b+s\right)\right]-r\left(e_{d}+e_{\mathrm{rd}}-se_{\mathrm{rd}}\right)+$ $\tau b_{1}\lambda_{2}\left(b-A\right)+c_{2}\lambda_{2}\left(w-c\gamma +w\gamma +A\tau -b\tau \right)\}$;.

$m_{1}=a+dk-dk\beta -a\rho +re_{dr}$;
$m_{2}=w-c\gamma +w\gamma +A\tau -b\tau +x\gamma \tau$.
$m_{3}=-c+w-b\tau +x\tau$;
$m_{4}=c_{2}\left(1+\lambda_{1}\right)\left(c_{1}-c_{2}\lambda_{2}\right)$; and
$m_{5}=\tau c_{1}\left(A-b\right)+\left(r-rs\right)e_{d}$.

In fact, when participants make price decisions, they cannot get all the market information of their competitors, nor can they adjust their price strategies with limited rationality. Decision makers show bounded rational behaviors and make the next decisions according to the current marginal utility. When the marginal utility of the current period is positive, decision makers will increase the speed of price adjustment in the next period, otherwise they will reduce the speed of price adjustment in the next period. The discrete dynamic game model of a low-carbon, closed-loop supply chain considering return rates can be described as follows:

(7) $$\{\begin{array}{c} p_{d}\left(t+1\right)=p_{d}\left(t\right)+\alpha_{1}p_{d}\left(t\right)\frac{\partial U_{m}\left(t\right)}{\partial p_{d}\left(t\right)}\\ p_{r}\left(t+1\right)=p_{r}\left(t\right)+\alpha_{2}p_{r}\left(t\right)\frac{\partial U_{r}\left(t\right)}{\partial p_{r}\left(t\right)} \end{array},$$

where $\alpha_{1}\;\mathrm{and}\;\alpha_{2}$ represent the price adjustment speeds of the manufacturer and retailer.

## 3. The Local Stability of Dynamic System (7)

### 3.1. Equilibrium Points

When $p_{d}\left(t+1\right)=p_{d}\left(t\right)\;\mathrm{and}\;p_{r}\left(t+1\right)=p_{r}\left(t\right)$, the four equilibrium solutions can be obtained:

$E_{1}=\left(0,\;0\right);$
$E_{2}=\left(\frac{n_{1}+b_{1}\left[\mu \left(c+b\tau -x\tau \right)+\tau \lambda_{2}\left(b-A\right)\right]+c_{2}\left[\mu \left(-c+w-b\tau +x\tau \right)+\lambda_{2}m_{3}\right]}{2b_{1}},\;0\right);$
$E_{3}=\left(0,\;\frac{\left(1+\lambda_{1}\right)\left[m_{1}-\tau c_{1}\left(A-b\right)-e_{d}r\left(1-s\right)\right]+b_{2}\left(w+A\tau -b\tau +\lambda_{1}n_{0}\right)}{2b_{2}\left(1+\lambda_{1}\right)}\right);$
$E_{4}=\left(p_{d}^{*},\;p_{r}^{*}\right);$

where $n_{0}=-c+2w+\tau \left(A-b+x\right)$ and $n_{1}=dk\beta +a\rho -re_{d}+re_{rd}-rse_{rd}$.

Economically, it makes no sense for the manufacturer and retailer to have zero prices, so the features of $E_{1}$, $E_{2}$, and $E_{3}$ were not studied. Next, we only considered the stability of the Nash equilibrium point ($E_{4}$), and the Jacobian matrix of dynamic System (7) is given as follows:

(8) $$J\left(p_{d}^{*},\;p_{r}^{*}\right)=\left|\begin{array}{cc} 1+\alpha_{1}f_{1} & \alpha_{1}\left(c_{1}-c_{2}\lambda_{2}\right)p_{d}^{*}\\ \alpha_{2}c_{2}\left(1+\lambda_{1}\right)p_{r}^{*} & 1+\alpha_{2}f_{2} \end{array}\right|,$$

where

$f_{1}=n_{1}+c_{1}p_{r}^{*}+\mu b_{1}\left(c+b\tau -x\tau \right)+\tau b_{1}\lambda_{2}\left(-A+b\right)+\mu c_{2}\left(n_{0}-\tau A\right)+c_{2}\lambda_{2}m_{3},$
$f_{2}=\left(1+\lambda_{1}\right)\left[m_{1}-m_{5}-4b_{2}p_{r}^{*}\right]+b_{2}\left(w+A\tau -b\tau \right)+b_{2}\lambda_{1}n_{0}.$

The corresponding characteristic polynomial of dynamic System (7) can be written as follows:

$$f\left(\lambda \right)=\lambda^{2}+M\lambda +N,$$

where M = 2 + $\alpha_{1}f_{1}+\alpha_{2}f_{2}$ and N = ($1+\alpha_{1}f_{1})$($1+\alpha_{2}f_{2})-\left[\alpha_{1}\left(c_{1}-c_{2}\lambda_{2}\right)p_{d}^{*}\right]$[$\alpha_{2}c_{2}\left(1+\lambda_{1}\right)p_{r}^{*}]$.

M and N represent the trace and determinant of the Jacobian matrix J($p_{d}^{*},\;p_{r}^{*})$, respectively.

The eigenvalues of the Jacobian matrix at the corresponding equilibrium point determine system stability. According to the Routh–Hurwitz condition, when the nonzero eigenvalues of the equilibrium point are all less than one, dynamic System (7) will be in a stable state; when one of the nonzero eigenvalues of the equilibrium point are larger than one, dynamic System (7) will be in an unstable state. The stable conditions of dynamic System (7) must satisfy the following conditions:

(9) $$\{\begin{array}{l} F\left(1\right)=1-M+N>0\\ F\left(-1\right)=1+M+N>0\\ F\left(0\right)=1-\left|N\right|>0 \end{array}.$$

Solving the inequality Equation (9), we can obtain the stable region of dynamic System (7). In the stable region, dynamic System (7) is locally stable with the initial values of prices within a certain range. Because these limitations are very complex, solving inequality Equation (9) is very complicated. Next, we give the stable region of dynamic System (7) through numerical simulation.

According to the current situation and characteristics of the low-carbon, closed-loop supply chain, we set parameter values as follows: $a=200,\;w=20,\;\rho =0.6,\;\beta =0.6,\;b_{1}=3,b_{2}=2,c_{1}=c_{2}=0.5,$ $e_{d}=30,\;e_{dr}=e_{rd}=1,d=5,k=12,s=0.4,\;r=0.7,\;\varepsilon =5,\;m=6,\mu =0.5,\;h_{1}=6,\;h_{2}=8,$ $\lambda_{1}=0.2,\;\lambda_{2}=0.2,\;\gamma =0.8,\;A=2,b=3,\;\tau =0.4,\;x=4,\;\mathrm{and}\;c=12$.

According to the parameter values above, we can obtain $E_{4}=\left(\;28.8,\;37.28\right)$. The Jaobian matrix is:

(10) $$J\left(E_{4}\right)=\left|\begin{array}{cc} 1-172.8\alpha_{1} & 11.52a_{1}\\ 22.37a_{2} & 1-178.9a_{2} \end{array}\right|.$$

The characteristic equation of Jacobian Matrix (10) is:

$$f\left(\lambda \right)=\lambda^{2}+M\lambda +N,$$

where $M=2-172.8\alpha_{1}-178.98\alpha_{2}$, and $N=\left(1-172.8\alpha_{1}\right)\left(1-178.98\alpha_{2}\right)-257.6\alpha_{1}\alpha_{2}$.

**Figure 2:**
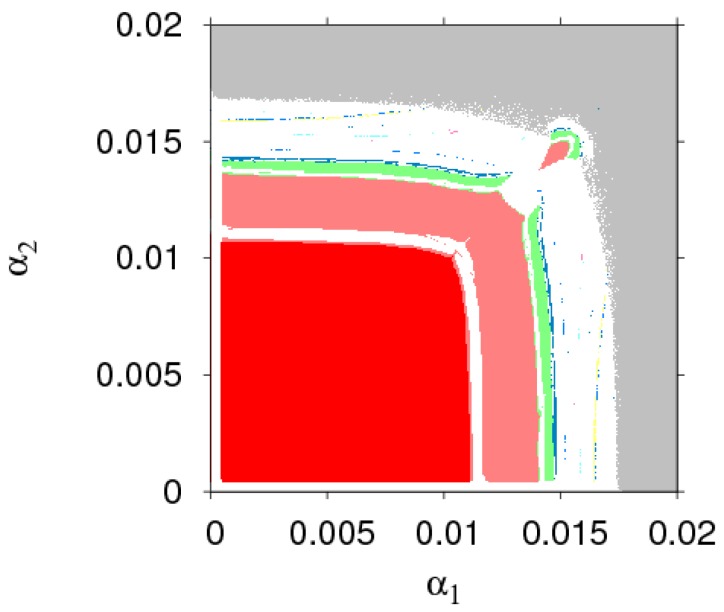
The parameter basin of dynamic System (7) in the ($\alpha_{1},\;\alpha_{2}$) plane.

The parameter basin is a powerful tool for numerical simulation, which can show the evolution process of dynamic System (7) into a chaotic state. Based on the stability conditions in Equation (9), Figure 2 shows the route of dynamic System (7) to chaos. Different colors represent different periods, for example: stable (red), period-2 (pink), period-3 (yellow), period-4 (green), period-5 (black), period-7 (wine red), period-8 (blue), chaos (white), and divergence (grey). From Figure 2, we find that dynamic System (7) goes into chaos through period bifurcation with an increasing $\alpha_{1}$ or $\alpha_{2}$. If the price adjustment parameters are in the red region, dynamic System (7) is in a stable state. If the price adjustment parameters are in the grey region, the manufacture or the retailer will withdraw from the competitive market.

### 3.2. The Stable Region of Dynamic System (7) with Changing Parameters

**Figure 3:**
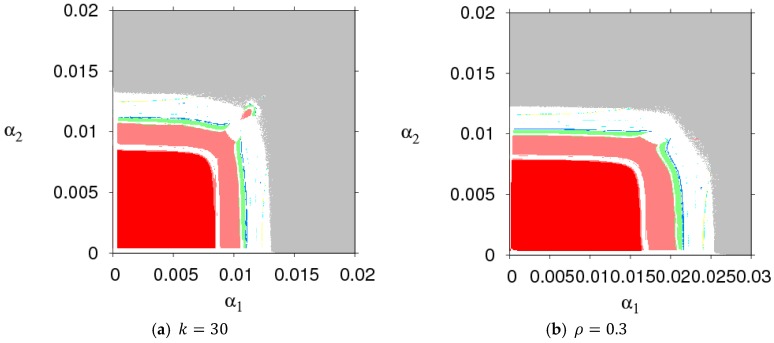

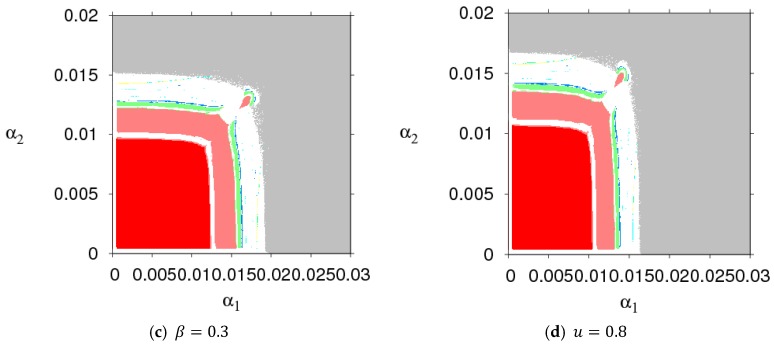
The parameter basins of dynamic System (7) under different parameter values.

Figure 3 shows the parameter basins of dynamic System (7) when k, ρ, β, and d take different values. Comparing the sizes of stability regions (red region) in Figure 2 and Figure 3, we found that a high level of CER decreased the stable region of dynamic System (7). An increasing customer loyalty to the direct marketing channel decreased the stable region of the manufacturer’s price adjustment and increased that of the retailer. An increasing μ decreased the stable region of the manufacturer’s price adjustment and had no effect on the stable range of the retailer’s price adjustment. Thus, the manufacturer and retailer should adjust parameters according to the actual market conditions so that dynamic System (7) is in a stable state.

**Figure 4:**
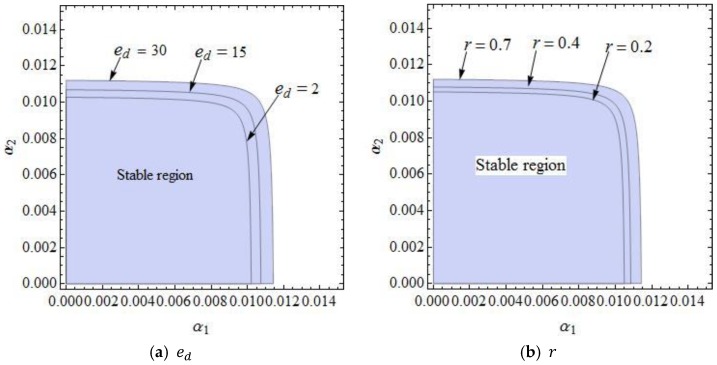

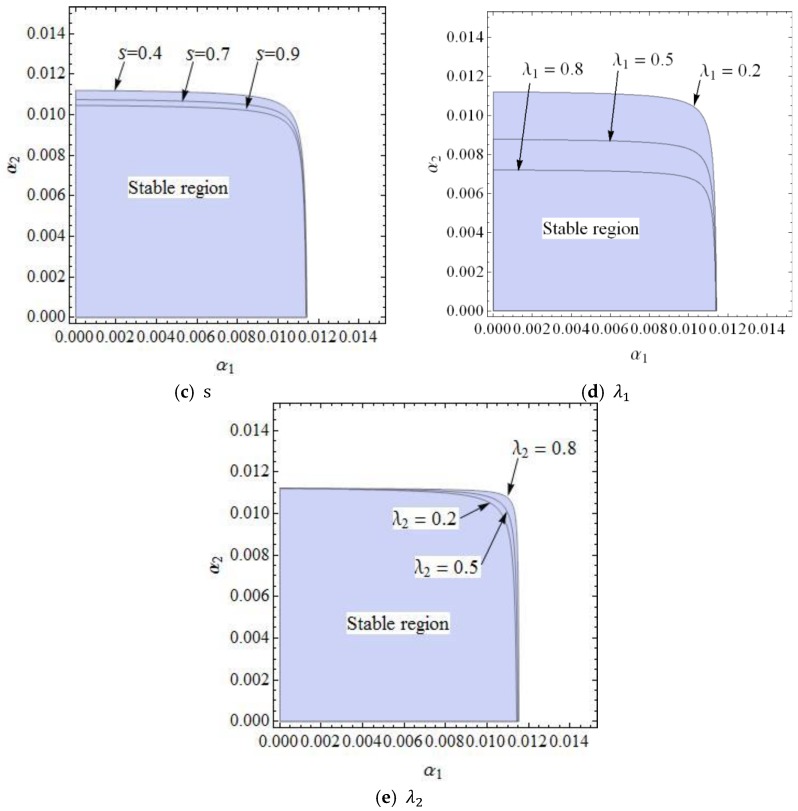
The parameter basins of $\alpha_{1}$ and $\alpha_{2}$ with different parameter values.

Figure 4 gives the effect that changing parameters had on the stability region of dynamic System (7). Figure 4a shows the stable regions of dynamic System (7) with $e_{d}$ having different values when other parameters are fixed. Stability regions of dynamic System (7) increased when $e_{d}=2,\;15,\;\mathrm{and}\;30$. Figure 4b shows that the stable regions of dynamic System (7) enlarged with different values of r. Figure 4c shows that the stable regions of dynamic System (7) shrank with different values of r. Figure 4d,e are the changes of stable regions with the change of $\lambda_{1}$ and $\lambda_{2}$; the scope of $\alpha_{2}$ decreased and that of $\alpha_{1}$ remained unchanged with the change of $\lambda_{1}$. Similarly, the stability regions of dynamic System (7) remained unchanged with $\lambda_{2}$ increasing (shown in Figure 4e). So dynamic System (7) was more sensitive to $\lambda_{1}$ than $\lambda_{2}$. The high level of fairness concerns of the retailer shrank the stability region of the Nash equilibrium point, while the fairness concern level of the manufacturer had little effect on system stability. The retailer should be more cautious on adjusting the price strategy than the manufacturer.

From the above analysis, we made some conclusions: (1) The increase of CER shrank the stability region of dynamic System (7), which weakened the market competition. The customers’ preference for the direct marketing channel decreased the stable range of price adjustment of the direct marketing channel and enlarged the stable range of price adjustment of the traditional channel. The change of share in the profit target of the manufacturer had no effect on the stable range of the retailer’s price adjustment, and it shrank that of the manufacturer’s price adjustment. (2) The fairness concern behavior of the manufacturer had little effect on the stability region of dynamic System (7), while the fairness concern of the retailer was disadvantageous in keeping dynamic System (7) in the stability region, which indicated the retailer’s fairness concern behavior had greater impact on the stability of dynamic System (7) than that of the manufacturer’s fairness concern behavior. (3) Raising the service level decreased the stable region of dynamic System (7), while the increase of return rate increased the stability region of dynamic System (7).

## 4. The Numerical Simulation of Dynamic System (7)

### 4.1. The Complex Entropy Analysis of Dynamic System (7) with the Price Adjustment Speed

**Figure 5:**
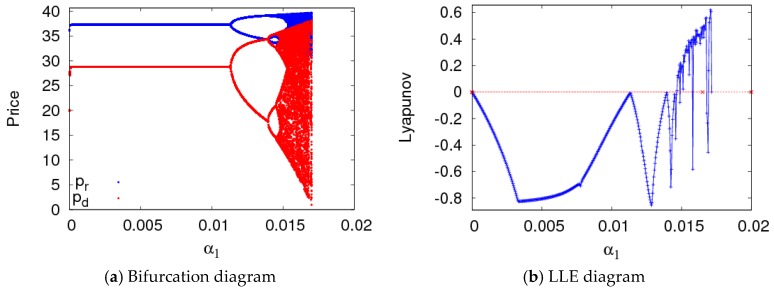
The features of dynamic System (7) with the change of $\alpha_{1}$ when $\alpha_{2}=0.008$.

In this section, parameter values were set the same as in the previous section, and the bifurcation diagram and entropy diagram of dynamic System (7) are shown in Figure 5, with $a_{1}$ varying and $a_{2}=0.008$. Dynamic System (7) lost its stability through flip bifurcation with an increasing $\alpha_{1}$, which was in accordance with Figure 2. Dynamic System (7) was in a stable state when $\alpha_{1}\in \left[0,\;0.0115\right]$, in the two-period state when $\alpha_{1}\in \left(0.0115,\;0.0135\right]$, and entered into chaos finally through flip bifurcation. The Largest Lyapunov Exponent (LLE)of dynamic System (7) is shown in Figure 5b when $\alpha_{1}\in \left[0,\;0.016\right]$. The LLE of dynamic System (7) was less thanzero when dynamic System (7) was in stable state. The LLE of dynamic System (7) was relatively large when dynamic System (7) was in chaotic state. Therefore, with an unreasonable change in the price adjustment parameter, the LLE of the system will increase, and the system will lose its stability. The manufacturer and retailer need to adjust price parameters very carefully in order to maintain system stability. 

**Figure 6:**
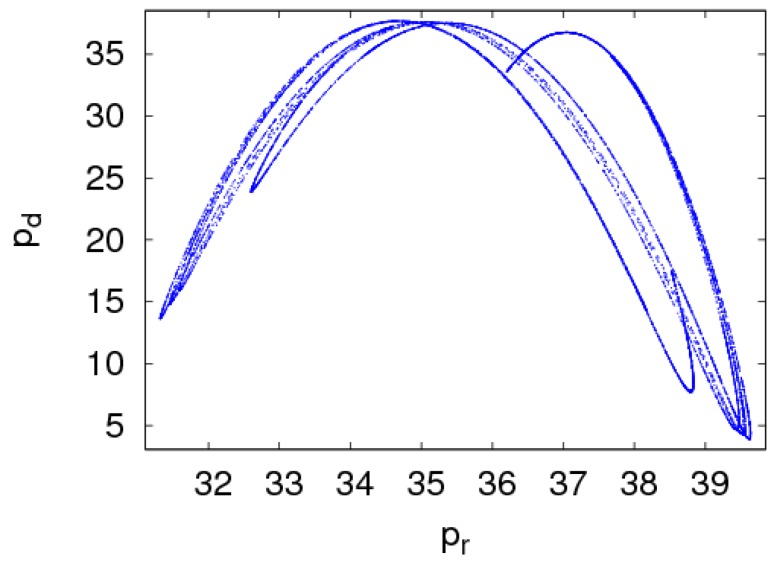
Chaos attractors of dynamic System (7) with $\alpha_{1}=0.0165$ and $\;\alpha_{2}=0.008$.

Chaotic attractors can reflect the motion characteristics of chaotic systems, and they are also a kind of chaotic steady-state motion pattern of chaotic systems. The chaotic attractors of dynamic System (7) are shown in Figure 6 when $\alpha_{1}=0.0165$ and $\alpha_{2}=0.008$. In the chaotic state, the prices of the manufacturer and retailer were in disorder. All the motions outside the chaotic attractors made dynamic System (7) tend to the attractor, and it belonged to the stable force; all the motions inside the chaotic attractors made dynamic System (7) repel the attractor, and it belonged to the unstable force.

### 4.2. The Dynamic Characteristics of Dynamic System (7) with Changing Parameters

In this section, the evolution characteristics of dynamic System (7) were analyzed with the change of $\rho ,\;\lambda_{1}$, and $e_{d}$ when dynamic System (7) was in stable and two-period states.

**Figure 7:**
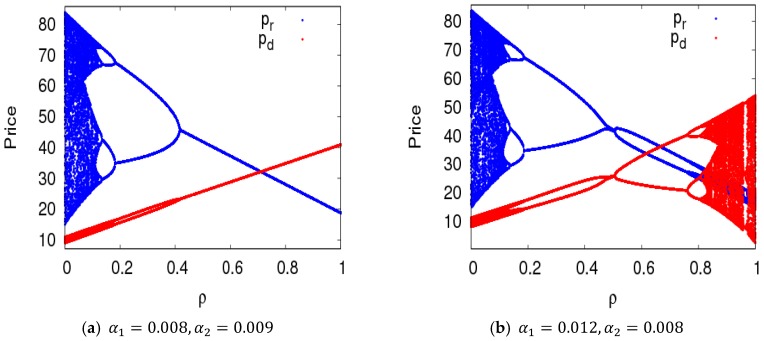
The evolution characteristics of dynamic System (7) when ρ∈[0, 1].

Dynamic System (7) was in a stable state with $\alpha_{1}=0.008,\alpha_{2}=0.009$, and with other parameters fixed as above. Figure 7a shows the price bifurcation diagram of dynamic System (7) with the change of ρ. When 0<ρ≤0.123, dynamic System (7) was in a chaotic state; when 0.123<ρ≤0.13, dynamic System (7) was in an eight-period state, four-period state when 0.13<ρ≤0.18, two-period state when 0.18<ρ≤0.42, and a stable state when ρ≥0.42. Figure 7b shows the price bifurcation diagram of dynamic System (7) with change of ρ when $\alpha_{1}=0.012\;\mathrm{and}\;\alpha_{2}=0.008$. The value range of ρ that kept dynamic System (7) in a stable state was very small.

**Figure 8:**
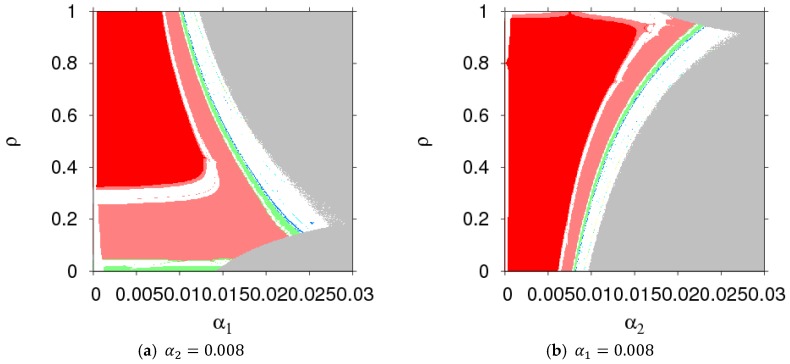
The parameter basins of dynamic System (7) in the planes of $\left(\alpha_{1},\;\rho \right)$ and $\left(\alpha_{2},\;\rho \right)$.

In order to better show the evolution process of dynamic System (7) with the change of ρ, we gave two-dimensional bifurcation graphs of dynamic System (7) in the plane of $\left(\alpha_{1},\;\rho \right)$ and $\left(\alpha_{2},\;\rho \right)$, which are shown in Figure 8. From Figure 8, we could clearly grasp the operating status of dynamic System (7) when the parameters ($\alpha_{1},\;\alpha_{2},\;\mathrm{and}\;\rho$) had different values.

**Figure 9:**
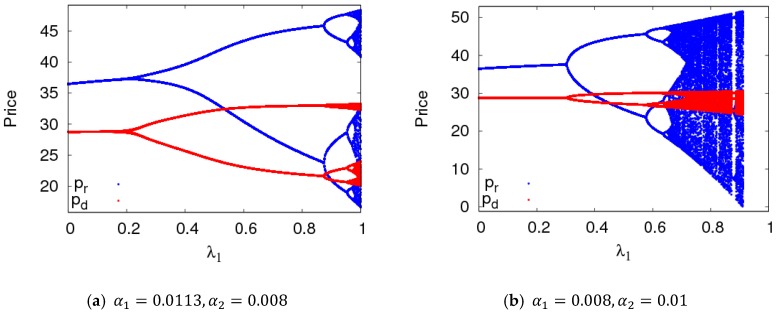
The evolution characteristics of dynamic System (7) when $\lambda_{1}\in \left[0,\;1\right]$.

Figure 9 shows the price bifurcation diagrams of dynamic System (7) with the change of $\lambda_{1}$. As can be seen from Figure 9, when $\alpha_{1}\;\mathrm{and}\;\alpha_{2}$ took different values, dynamic System (7) changed from a stable state to a doubling bifurcation period and finally to a chaotic state with an increasing $\lambda_{1}$.

**Figure 10:**
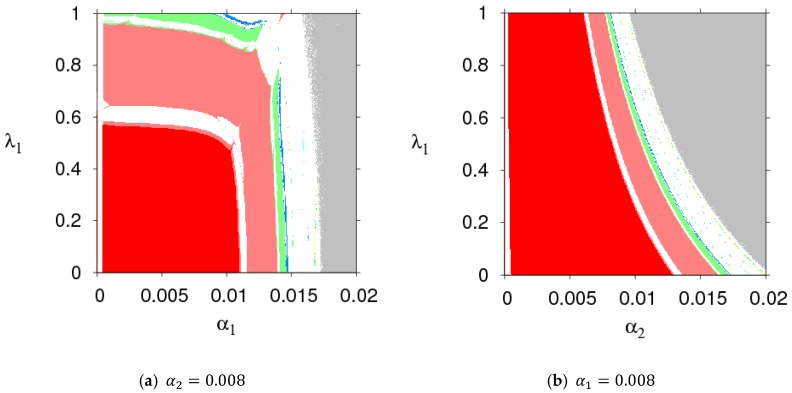
The parameter basins of dynamic System (7) in the planes of $\left(\alpha_{1},\;\lambda_{1}\right)$ and $\left(\alpha_{2},\;\lambda_{1}\right)$.

Figure 10 gives two-dimensional bifurcation graphs of dynamic System (7) in the plane of $\left(\alpha_{1},\;\lambda_{1}\right)$ and $\left(\alpha_{2},\;\lambda_{1}\right)$. From Figure 10a, when $\alpha_{1}$ and $\lambda_{1}$ were all in the red region, dynamic System (7) was in a stable state. After that, dynamic System (7) entered into a chaotic state with increases of $\alpha_{1}$ and $\lambda_{1}$. From Figure 10b, when $\alpha_{2}\leq 0.0062$, dynamic System (7) was in a stable state with the change of $\lambda_{1}$; when $\alpha_{2}>0.0062$, dynamic System (7) was in a flip bifurcation or chaotic state.

**Figure 11:**
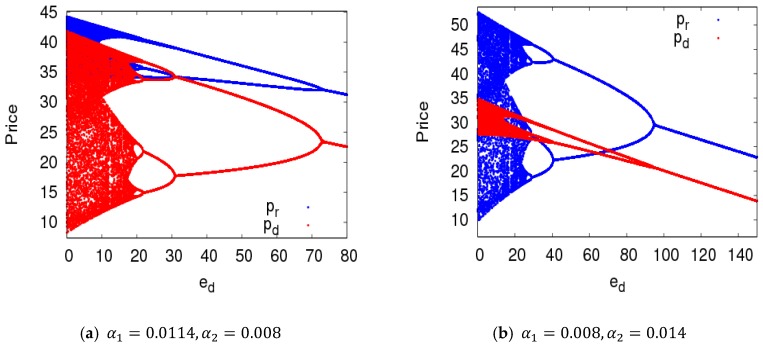
The evolution characteristics of dynamic System (7) with an increasing $e_{d}$.

Figure 11 shows the price bifurcation diagrams of dynamic System (7) with the change of $e_{d}$. We can see that when dynamic System (7) was in an unstable state, raising the sensitivity of customer demand to the return rate made dynamic System (7) return to a stable state from a chaotic state.

**Figure 12:**
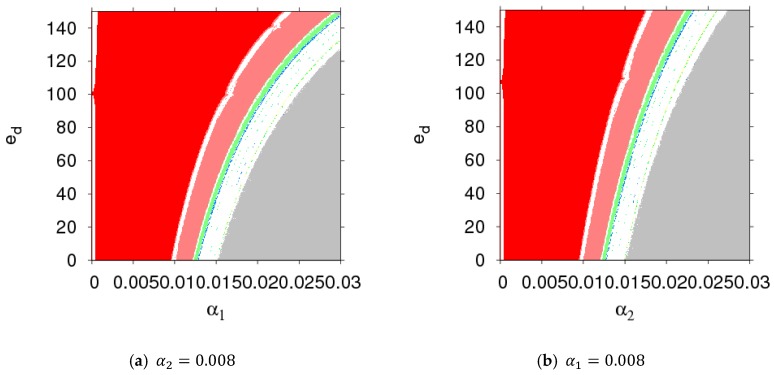
The parameter basins of dynamic System (7) in the planes of $\left(\alpha_{1},\;\lambda_{1}\right)$ and $\left(\alpha_{2},\;\lambda_{1}\right)$.

Figure 12 gives two-dimensional bifurcation graphs of dynamic System (7) in the plane of $\left(\alpha_{1},\;\lambda_{1}\right)$ and $\left(\alpha_{2},\;\lambda_{1}\right)$. From Figure 10a, when $\alpha_{1}$ and $\lambda_{1}$ were all in the red region, dynamic System (7) was in stable state. After that, dynamic System (7) entered into a chaotic state with increases of $\alpha_{1}$ and $\lambda_{1}$. From Figure 10b, when $\alpha_{2}\leq 0.0062$, dynamic System (7) was in stable state with the change of $\lambda_{1}$; when $\alpha_{2}>0.0062$, dynamic system (7) was in a flip bifurcation state or a chaotic state.

### 4.3. The Effects of Price Adjustment Speed on the Utilities of Dynamic System (7)

The stability of dynamic System (7) was affected by the change of parameter values. It was complicated for the manufacturer and retailer to make dynamic price decisions in an unstable state. So, we suspected that the utilities of the two sides were also seriously influenced. This section mainly analyzed the influence of changing parameters on the utilities of the manufacturer and retailer.

**Figure 13:**
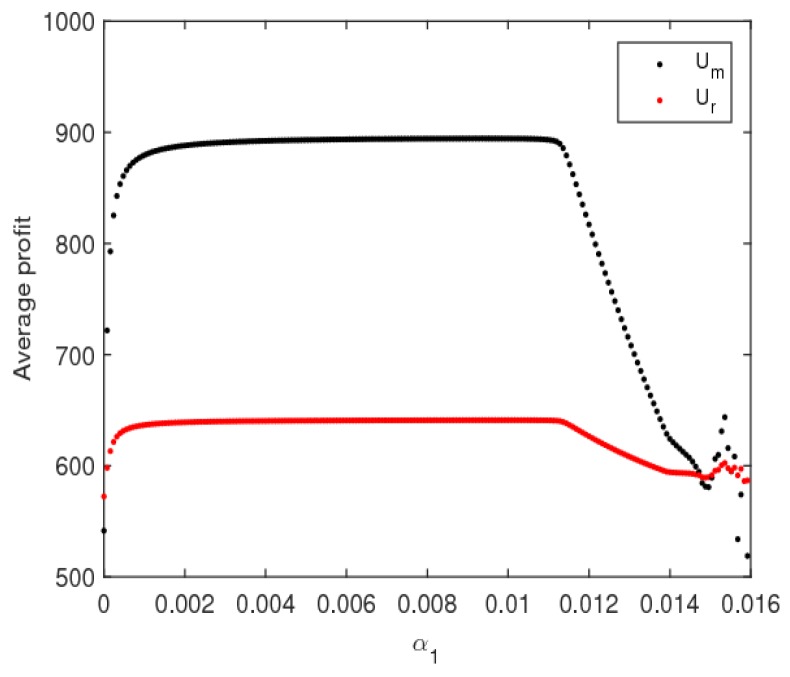
The average utilities of the manufacturer and retailer with an increasing $\alpha_{1}$.

Figure 13 is an evolution diagram of the average utilities of the manufacturer and retailer with the change of $\alpha_{1}$. In the periodic doubling bifurcation and chaotic states, the average utilities of the manufacturer and retailer all declined, while that of the retailer declined rapidly.

**Figure 14:**
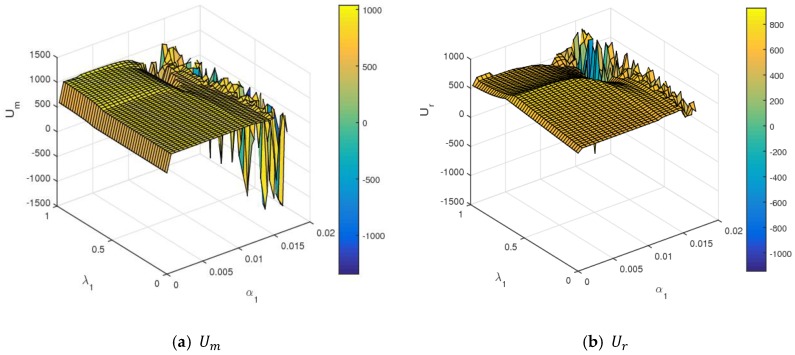
The utility changes of the manufacturer and retailer with respect to $\alpha_{1}$ and $\lambda_{1}$.

Figure 14 shows the influence of $\alpha_{1}$ and $\lambda_{1}$ on the utilities of the manufacturer and retailer using a three-dimensional grid. In Figure 14, when $\alpha_{1}$ and $\lambda_{1}$ were controlled in small values, the utilities of the manufacturer and retailer were almost stable. When $\alpha_{1}$ took a certain value, the utility of the manufacturer rose, and the utility of the retailer first increased and then decreased with an increasing $\lambda_{1}$. With a simulatneous increase of $\alpha_{1}$ and $\lambda_{1}$ values to the larger value range, dynamic System (7) became unstable and fell into chaos. With that, the utilities of the manufacturer and retailer changed violently and even had a great loss. So, the retailer should control the level of fariness concern to obtain maximum utility. In the chaotic state, it was very difficult to maximize profits and formulate long-term competitive strategies for the manufacturer and retailer in the competitive market.

**Figure 15:**
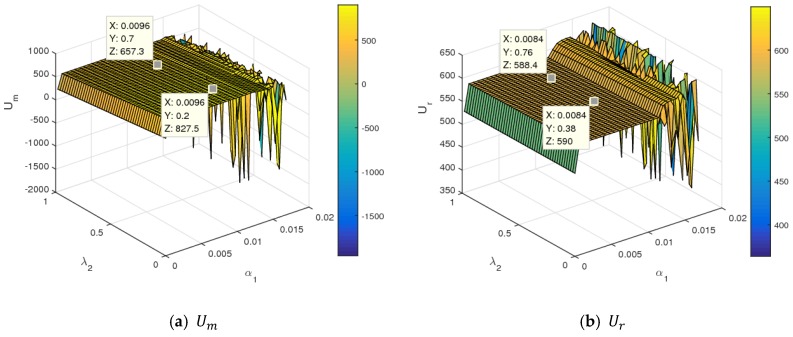
The utility changes of the manufacturer and retailer with respect to $\alpha_{1}$ and $\lambda_{2}$.

**Figure 16:**
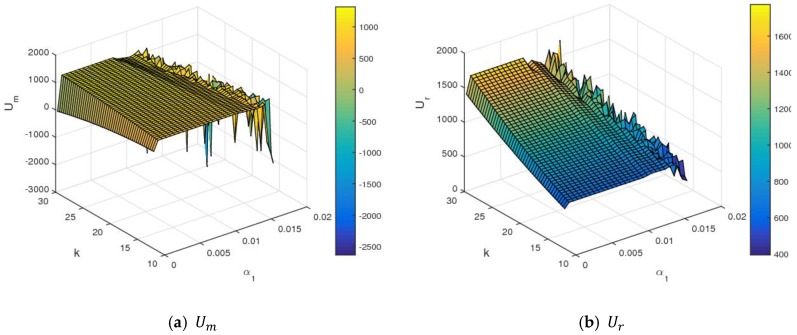
The utility changes of the manufacturer and retailer with respect to $\alpha_{1}$ and k.

**Figure 17:**
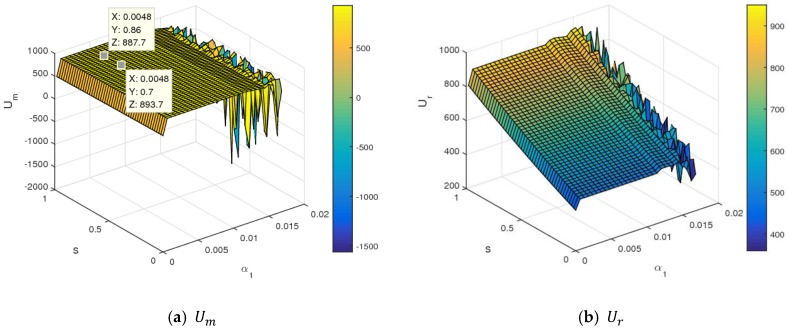
The utility changes of the manufacturer and retailer with respect to $\alpha_{1}$ and s.

**Figure 18:**
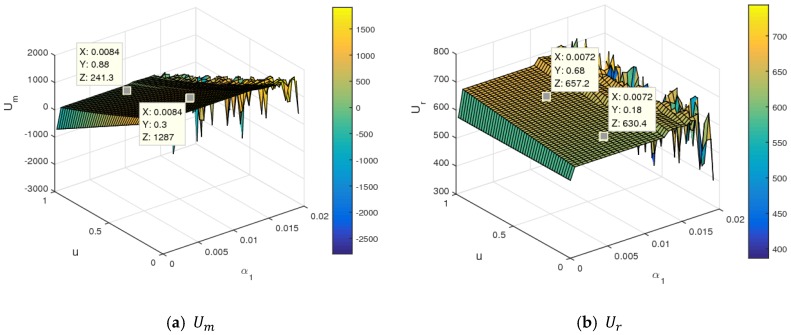
The utility changes of the manufacturer and retailer with respect to $\alpha_{1}$ and μ.

Figure 15 shows the utility changes of the manufacturer and retailer with $\alpha_{1}$ and $\lambda_{2}$ increasing simultaneously. From Figure 15, the manufacturer and retailer declined with an increase of $\lambda_{2}$, namely, the higher fariness concern behavior of the manufacturer was harmful for two sides to obtain maximum profit. With $\alpha_{1}$ and $\lambda_{2}$ increasing simultaneously to the larger value range, the utilities of the manufacturer and retailer changed violently and even had a great loss. Figure 16 shows the utility changes of the manufacturer and retailer with $\alpha_{1}$ and k increasing simultaneously. The utilities of the manufacturer and retailer increased with the increase of k. With the improvement of service input, the utility of the manufacturer decreased slightly, and that of retailer rose sharply, which is shown in Figure 17. Figure 18 shows that the utility of the manufacturer decreased sharply, and that of that retailer increased slightly with an increase in the proportion of the manufacturer’s profit share.

In summary, changes to parameter values had a great influence on the utilities of the manufacturer and retailer. In market competition, the manufacturer and retailer should pay attention to the parameter values. Choosing proper values for parameters is indispensable for the manufacturer and retailer to achieve business objectives.

## 5. Global Stability of Dynamic System (7)

Varying the parameters had a great influence on the stability of the system. The basins of attraction were an effective method for analyzing the effects of changing parameters on global stability, which included an attraction domain and an escaping area.

**Figure 19:**
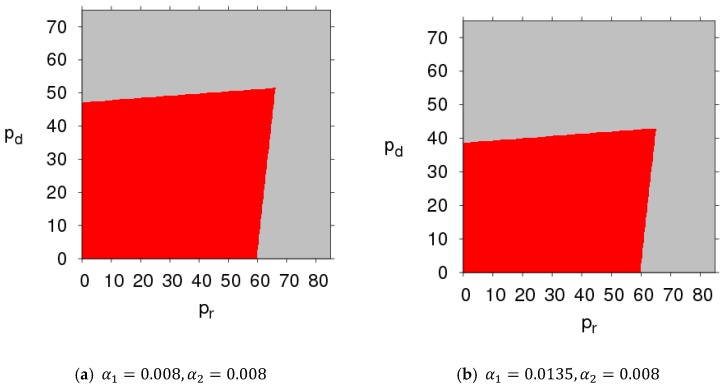

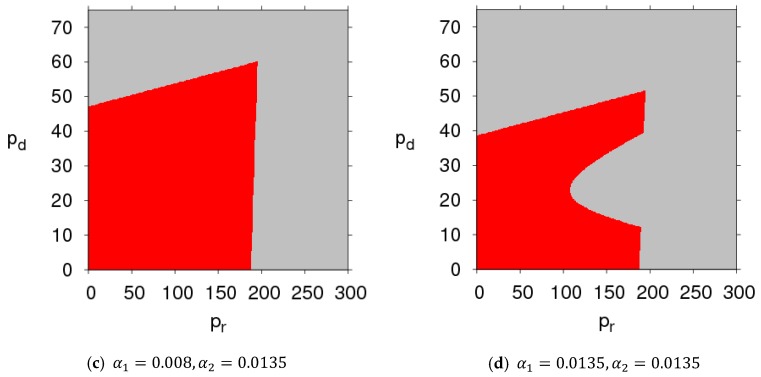
Basins of attraction of dynamic System (7).

By fixing the parameter values of dynamic System (7) as mentioned above, and setting different values for $\alpha_{1}$ and $\alpha_{2}$, respectively, the basins of attraction about initial prices $p_{d}$ and $p_{r}$ of dynamic System (7) were obtained (Figure 19; in which the red region denotes stable attraction domain and the grey region denotes escape area). We found that the attraction domain shrank with the increase of price adjustment parameters. From an economic point, the initial prices of the manufacturer and retailer should be in the basin of attraction in order to maintain market stability. The basin of attraction is the set of initial conditions. If the initial prices of $p_{d}$ and $p_{r}$ were taken from the attraction domain, the same attractor emerged from System (7) after a series of iterations. If the initial prices of $p_{d}$ and $p_{r}$ were outside the basins of attraction, dynamic System (7) fell into divergence.

## 6. Chaos Control

When the supply chain system was in chaos, the utilities of the manufacturer and retailer declined in an unstable state, which was harmful for all firms to obtain their business objectives. In order to reduce the risk caused by a chaotic state of the supply chain system, the decision makers should select suitable adjustment parameters to keep the supply chain system in a stable state or delay the supply chain system entering into a chaotic state. According to the above numerical simulation, if the parameter values or the price adjustment speeds were beyond the stable region, the supply chain system lost stability and even fell into chaos.

**Figure 20:**
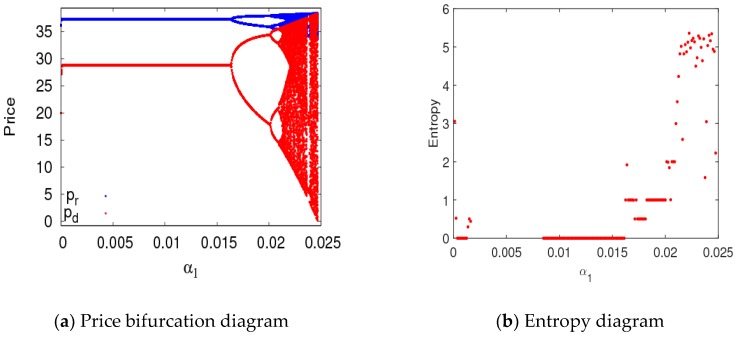
Bifurcation diagram and entropy of control System (11) with respect to $\alpha_{1}$ when v=0.3.

There are many methods to control the supply chain from the chaos state to the stable state such as the modified straight-line stabilization method [38], the time-delayed feedback method [39], and the OGY method [40]. In this section, the state feedback control method was used to delay or eliminate the chaos in the supply chain system. The controlled system is represented as follows:

(11) $$\{\begin{array}{c} \;p_{d}\left(t+1\right)=\left(1-v\right)p_{d}\left(t\right)+\alpha_{1}p_{d}\left(t\right)\frac{\partial U_{m}\left(t\right)}{\partial p_{d}\left(t\right)}+vp_{d}\left(t\right)\\ p_{r}\left(t+1\right)=\left(1-v\right)p_{r}\left(t\right)+\alpha_{2}p_{r}\left(t\right)\frac{\partial U_{r}\left(t\right)}{\partial p_{r}\left(t\right)}+vp_{r}\left(t\right) \end{array},$$

where v can be considered as the learning ability, adjustment ability, or adaptability of the manufacturer and retailer. For example, the manufacturer and retailer can adjust price or service input and CER through market research or by analyzing historical information. As what can be seen from Figure 20, chaos in control System (11) was delayed when v=0.3, and the first bifurcation in control System (11) occurred when $a_{1}=0.0168$. In the stable state, the system’s entropy was equal to zero; in the chaotic state, the system’s entropy approximately increased to five. Therefore, the manufacturer and retailer can delay the occurrence of price bifurcation in dynamic System (7) by choosing appropriate control parameters.

**Figure 21:**
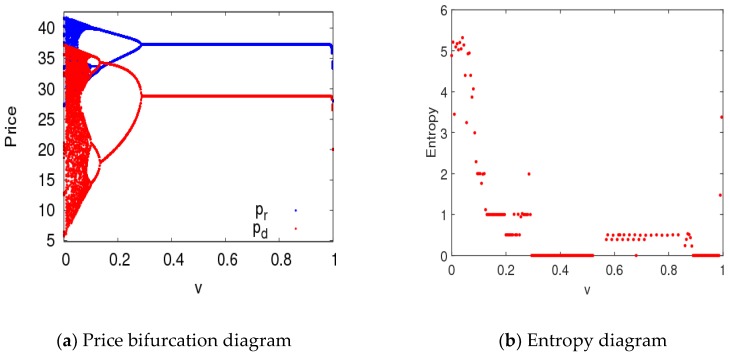
Evolution process and entropy of control System (11) with v∈(0, 1) when $\alpha_{1}=0.0156\;\mathrm{and}\;\alpha_{2}=0.008$.

Figure 21 depicts the evolution process and entropy of controlled System (11) with the change of v when $a_{1}=0.0156\;\mathrm{and}\;a_{2}=0.008$. Controlled System (11) was in an unstable state and had large entropy when v≤0.27; when 0.27<v≤1, controlled System (11) returned to the stable state. Therefore, when the dynamic system was in a chaotic state, the manufacturer and retailer can select appropriate control parameters to restore the competitive market to a stable state.

## 7. Conclusions

In this paper, we developed a dynamic game model of a low-carbon, closed-loop supply chain system in which the manufacturer had fair concern behaviors, fair CER, and made market share and profit maximization their objective. In addition, the retailer showed fair concern behaviors in market competition and provided service input to reduce return rate. The retailer recycled old products from customers and sold them to the manufacturer, the manufacturer remanufactured the recycled old products. Dynamic behaviors of the dynamic game model were analyzed using bifurcation, basin of attraction, chaotic attractors, and so on. The utilities of the manufacturer and retailer were described when parameters changed. The state feedback control method was used to control the chaos of the dynamic system. The following conclusions can be obtained.

(1) An increasing customer loyalty to the direct marketing channel will decrease the stable region of the manufacturer’s price adjustment and increase that of the retailer. The stable region of the system shrinks with an increasing CER level and retailer service level, which expands with return rates. 

(2) The dynamic system enters into chaos through flip bifurcation with an increase in price adjustment speed. In the stable state, the utilities of the manufacturer and retailer increase with the increase of the retailer’s fair concern, while utilities decrease with the increase of the manufacturer’s fair concern. The level of CER is beneficial for utility acquisition of the manufacturer and retailer. The retailer’s service level and the balance coefficient of the manufacturer make the utility of the manufacturer decline and that of the retailer increase. In the chaotic state, the average utility of the manufacturer and retailer all decline, while that of the retailer declines even more.

(3) By selecting appropriate control parameters, the dynamic system can return to a stable state from chaos again.

The research of this paper is of great significance to the participants’ price decision-making and supply chain operation management.
